# Expansion of the Spinocerebellar Ataxia Type 10 (SCA10) Repeat in a Patient with Sioux Native American Ancestry

**DOI:** 10.1371/journal.pone.0081342

**Published:** 2013-11-20

**Authors:** Khalaf Bushara, Matthew Bower, Jilin Liu, Karen N. McFarland, Ivette Landrian, Diane Hutter, Hélio A. G. Teive, Astrid Rasmussen, Connie J. Mulligan, Tetsuo Ashizawa

**Affiliations:** 1 Department of Neurology, University of Minnesota Medical Center, Minneapolis, Minnesota, United States of America; 2 Institute of Human Genetics, University of Minnesota Medical Center, Minneapolis, Minnesota, United States of America; 3 Department of Neurology and The McKnight Brain Institute, University of Florida, Gainesville, Florida, United States of America; 4 Movement Disorders Unit, Neurology Service, Hospital de Clínicas, Federal University of Paraná, Centro, Curitiba, Brazil; 5 Arthritis and Clinical Immunology Research Program, Oklahoma Medical Research Foundation, Oklahoma City, Oklahoma, United States of America; 6 Department of Anthropology and Genetics Institute, University of Florida, Gainesville, Florida, United States of America; University of Texas MD Anderson Cancer Center, United States of American

## Abstract

Spinocerebellar ataxia type 10 (SCA10), an autosomal dominant cerebellar ataxia, is caused by the expansion of the non-coding ATTCT pentanucleotide repeat in the *ATAXIN 10* gene. To date, all cases of SCA10 are restricted to patients with ancestral ties to Latin American countries. Here, we report on a SCA10 patient with Sioux Native American ancestry and no reported Hispanic or Latino heritage. Neurological exam findings revealed impaired gait with mild, age-consistent cerebellar atrophy and no evidence of epileptic seizures. The age at onset for this patient, at 83 years of age, is the latest documented for SCA10 patients and is suggestive of a reduced penetrance allele in his family. Southern blot analysis showed an SCA10 expanded allele of 1400 repeats. Established SNPs surrounding the SCA10 locus showed a disease haplotype consistent with the previously described “SCA10 haplotype”. This case suggests that the SCA10 expansion represents an early mutation event that possibly occurred during the initial peopling of the Americas.

## Introduction

Spinocerebellar ataxia type 10 (SCA10; OMIM#603516) is an autosomal dominant cerebellar ataxia variably associated with epilepsy and other nervous system disorders [1], [2]. The SCA10 mutation is an unstable expansion of an (ATTCT)n repeat in intron 9 of *ATAXIN 10* (*ATXN10*; NCBI GeneID: 25814; Genomic DNA Accession: NG_016212.1) on chromosome 22q. The polymorphic repeat expands up to 4,500 repeats in SCA10 patients [3] (normal range: ≤32 [4]; reduced penetrance range: 280–850 repeats [5]–[7]). All reported SCA10 cases occur in patients from Latin America with oral family histories, and in most cases physical characteristics, of Amerindian ancestry [1], [2], [8]–[13]. Thus, the SCA10 mutation was believed to have arisen among Amerindian populations south of the US-Mexican border. We report a patient of Sioux Indian descent, from Minnesota, with a very late-onset ataxia and an expanded ATTCT repeat. This result indicates that the SCA10 mutation is present in Native Amerindian populations in North America and suggests that the mutation may have evolved early in the process that first led to the peopling of the Americas.

## Results

### Neurological Exam and History

An 89 year-old man first noted balance loss at 83 years of age. The patient was clinically diagnosed to have Parkinson's disease by a local neurologist because he had “parkinsonian” gait with shuffling and difficulties in the initiation and balance although he had no tremor. He had no improvement with anti-parkinsonian medications. At age 86, he was evaluated for unstable gait at the University of Minnesota Ataxia Clinic.

His past medical history was significant for hypertension and mild congestive heart failure. He takes diltiazem, clonidine, potassium chloride, frosemide, aspirin and calcium supplement. He has never had any incidence suggestive of seizure or syncope. He is a previous heavy smoker for 40 years until 30 years ago and he drinks a glass of wine per week. The patient is a World War II veteran and retired attorney, and engaged in active life style.

The patient’s paternal ancestry is French and Irish, while his maternal ancestry is French and Sioux Indian. The patient’s maternal grandmother, a Sioux Indian, was noted to be part of the late 1800’s travelling show “Buffalo Bill’s Wild West.” The patient specifically reported no Hispanic, Latino, Spanish or Portuguese ancestry. The patient reported a distant maternal relative (half second cousin) who developed “balance problems” in her 70’s. Given the distance of the relationship, no other details were available about her diagnosis. The patient’s mother and father both survived into their 90’s with no evidence of neurologic disease. The maternal grandmother with Sioux Indian ancestry was also noted to have survived into her late 90’s with no evidence of neurologic disease.

On physical examination, his blood pressure was 150/67 mmHg, heart rate 69/minute and respiration rate 20/minute. General physical examination showed no dysmorphism. His mental status including short-term memory was intact. Speech was clear without dysarthria, and the central language processing was normal.

Cranial nerves were unremarkable except for interrupted pursuit eye movements. Saccadic initiation and velocity were normal. No nystagmus or other ocular motility abnormalities were detected. On motor examination, strength was within normal limits. There was no muscle atrophy, fasciculation, rigidity, or involuntary movements. Sensory examination showed distal shading of pin prick sensation in the lower extremities, but was intact to light touch and temperature in all extremities. Vibratory and position sensation was normal. Reflexes were symmetrically diminished in the upper and lower extremities and absent at the ankles. Coordination was intact to the finger-nose-finger test, but the heel-to-shin test was slightly impaired with mild dysmetria. There was no dysdiadochokinesia. In walking, he had a moderately stooped posture with a wide-based short-step gait with minimally decreased arm swing. Patient was unable to tandem walk.

MRI of the brain showed mild generalized cerebral atrophy, white matter ischemic changes and an old left parietal small infarct. No disproportional cerebellar atrophy was noted. Electroencephalogram was normal. Electromyography and nerve conduction studies showed moderate, predominantly axonal, sensorimotor peripheral neuropathy.

### SCA10 expansion sizing and SNP haplotypes surrounding the SCA10 locus

**Figure 1:**
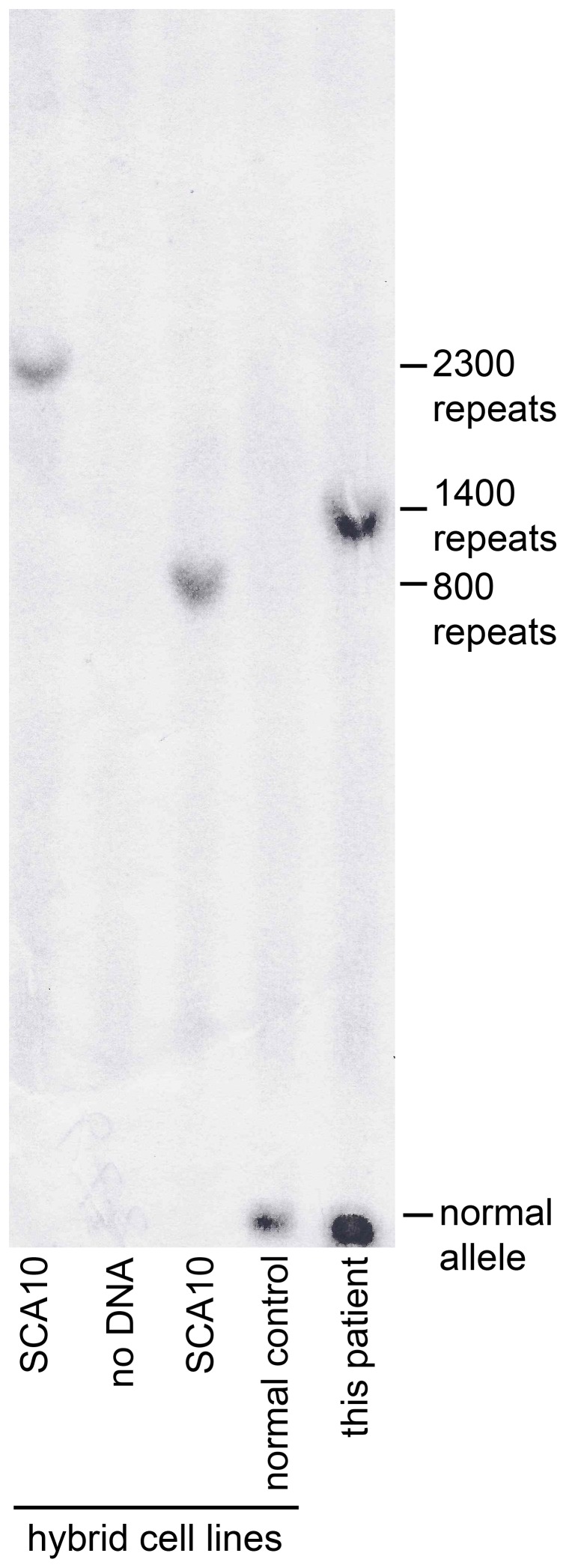
Southern blot analysis of the SCA10 ATTCT repeat expansion in our Sioux patient with SCA10. Lane 1: positive control, 2300 repeats (genomic DNA from SCA10 somatic cell hybrid line (SCH)) [30]; Lane 2: no DNA control; Lane 3: positive control, 800 repeats (genomic DNA from SCA10 SCH); Lane 4: negative control (genomic DNA from normal control SCH); Lane 5: DNA from Sioux SCA10 patient.

Southern blot analysis revealed an SCA10 expansion of 1400 repeats in this patient (Figure 1).

We examined SNPs surrounding the SCA10 locus (Table 1) and found a haplotype in this individual that is consistent with the previous described “SCA10 haplotype” [14].

**Table 1:** Haplotype analysis of single nucleotide polymorphisms (SNPs) surrounding the SCA10 locus in the Sioux SCA10 patient.

SNP ID&	HGVS nomenclature	Distance from SCA10 expansion†	SNP alleles	Sioux SCA10	Brazilian/Mexican SCA10*	SCA10 haplotypê
rs136002	NC_000022.10:g.46189190G>A	–2045	A/G	A/G	A	NR
rs5765626	NC_000022.10:g.46189278G>A	–1957	A/G	G	G	NR
rs5764850	NC_000022.10:g.46190037A>C	–1198	C/A	C/A	C	C
rs136003	NC_000022.10:g.46190341_46190342insA	–898	-/A	-	-	NR
**SCA10**		**0**	**---**	**1400**	**EXP**	**EXP**
rs72556348	NC_000022.10:g.46191352G>A	47	A/G	G	G	G
rs72556349	NC_000022.10:g.46191608G>A	303	A/G	G	G	G
rs72556350	NC_000022.10:g.46191675C>T	370	C/T	C	C	C
rs136005	NC_000022.10:g.46192395T>C	1091	C/T	**C**/T^$^	C	NR
rs9614518	NC_000022.10:g.46192600A>T	1296	A/T	A	A	NR
rs6006808	NC_000022.10:g.46192642G>A	1338	A/G	G	G	NR
rs11912672	NC_000022.10:g.46192880A>G	1576	A/G	A	A	NR
rs9614781	NC_000022.10:g.46192942C>G	1638	C/G	C	C	NR

& SNPs used in this study were originally studied in Almeida et al [14]. †Distance of the SNP is relative to the SCA10 expansion and is expressed in base pairs. Locations upstream and downstream of the SCA10 expansion are denoted by negative and positive values, respectively. *, The common disease haplotype of Mexican and Brazilian families in our SCA10 cohort of 31 families [29]. ?The “SCA10 haplotype” originally described in Almeida et al [14]. NR, not reported by Ameida et al [14], although these SNPs are mentioned by this study. ^$^, “C” allele segregates with SCA10 expansion. No additional sequence changes were seen outside of the SNPs reported.

## Discussion

**Figure 2:**
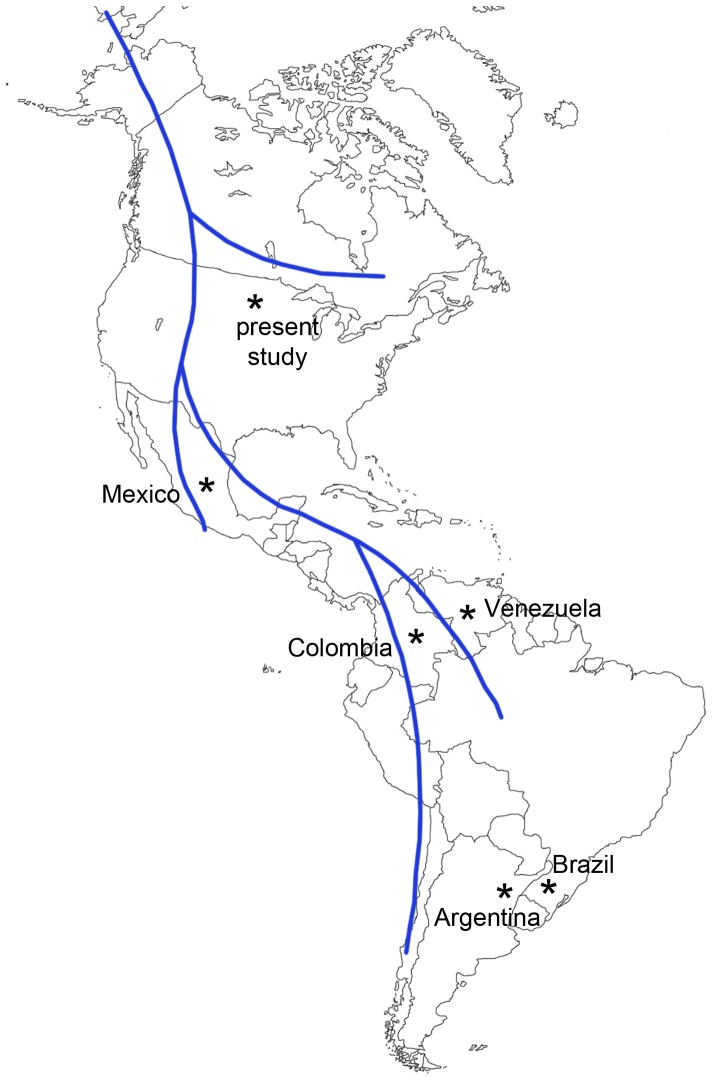
The distribution of SCA10 in the American continents and the proposed dispersal pattern of the mutation. Possible dispersal patterns of Native American and Amerindian populations as they began entering the Americas ∼15,000 years ago are shown as solid blue lines. Asterisks indicate countries where SCA10 patients have documented ancestral ties.

SCA10 has been found in patients from Mexico, Brazil, Argentina, Colombia and Venezuela [1], [8]–[13]. Several searches for SCA10 expansions in patients with ataxia inherited in an autosomal dominant fashion failed to identify the expanded ATTCT repeat allele in other countries including Italy [15], France [16], Poland [17], Portugal [18] and China [19]–[21]. Thus, SCA10 is believed to be extremely rare, or non-existent, outside of Latin American populations and SCA10 patients identified to date report oral histories of Amerindian ancestry [1], [2]. These observations, combined with the relatively wide geographic distribution of SCA10 throughout Latin American countries, have led to the hypothesis of a founder effect mutation that likely arose in an ancestral Amerindian population [14], [22]. Population and molecular genetic data support the hypothesis that Amerindian and Native American ancestors migrated from east central Asia across the exposed Bering land bridge to North America, and then spread throughout the Americas from north to south [23]–[26]. Furthermore, there is evidence that the migrating population experienced a long period of population isolation, possibly in Beringia, during which time numerous genetic variants evolved that are found only in the Americas where they are spread throughout North and South America [27]. This period of population isolation and genetic diversification is estimated to have lasted at least 7,000–15,000 years [28]. In this context, the existence of the expanded SCA10 allele in the individual of Sioux Indian ancestry suggests that the expansion of the ATTCT repeat may have evolved during the period of population isolation that ancestral Native Americans experienced prior to migration throughout the Americas (Figure 2).

This patient was nearly asymptomatic until his 80s despite the expansion size of 1,400 repeats, which is considered to be a full mutation allele. The onset of this patient’s disease is the latest described so far for SCA10. Had he not lived beyond his age at onset (which exceeds the average lifespan of North American males), his expanded allele would have been considered to be a reduced penetrance allele. Furthermore, he has no affected family members, further supporting the possibility of late onset or reduced penetrance of the SCA10 repeat expansion in this family, unless this is a *de novo* mutation case, which would be extremely rare. The mechanism of this reduced virulence of this patient’s SCA10 mutation remains unknown although the mechanism may be working either in *cis* or *trans* as suggested for other reduced penetrant expansions [5]–[7]. Our case suggests that the original SCA10 mutation is likely to have occurred early in the peopling of the Americas, before the southward migration to present-day Latin America, and possibly prior to their entry to the Americas.

## Materials and Methods

### Ethics Statement

This work was conducted under a protocol approved by the Institutional Review Board of the University of Minnesota Medical School. Blood samples were drawn after written informed consent was obtained.

### Neurological Exam and Family History

A detailed neurological exam was performed and history was collected.

### Southern Blot for SCA10 expansion

High molecular weight DNA was extracted with conventional methods for peripheral blood leucocytes. Genomic DNA was digested with *EcoRI*, subjected to 0.8% agarose gel electrophoresis, followed by Southern blot analysis using a ^32^P-labelled probe as described previously [3] with minor modifications.

### SCA10 haplotyping

Haplotype analysis was performed using PCR primers for single nucleotide polymorphisms (SNPs). PCR primers and conditions for these SNPs were described in [14]. These SNPs define an “SCA10 haplotype” and surround the SCA10 expansion. PCR products were purified and subjected to Sanger sequencing at the Interdisciplinary Center for Biotechnology Research sequencing core at the University of Florida. SNPs were identified by examining the electropherogram for each sequencing reaction. To identify the SCA10 haplotype for SNP, rs136005, that segregates with the normal allele, a 1.5-Kbp DNA fragment containing the normal ATTCT repeat allele was PCR amplified using the forward primer from the flanking PCR reaction used to size normal SCA10 alleles and is located upstream of the ATTCT repeat [29] and a reverse primer for SNP rs136005, located downstream of the expansion. PCR conditions were such that only the 1.5 kb fragment containing the wild-type allele was amplified while the larger 8.5 kb SCA10 allele associated with the disease was not amplified (data not shown). The 1.5 kb fragment containing the normal allele was purified and subjected to sequence analysis using the same primers the forward and reverse PCR primers for SNP, rs136005.
